# National audit of post-operative management in spinal surgery

**DOI:** 10.1186/1471-2474-7-47

**Published:** 2006-05-31

**Authors:** Alison H McGregor, Ben Dicken, Konrad Jamrozik

**Affiliations:** 1 Biosurgery & Surgical Technology, Faculty of Medicine, Imperial College, London, UK; 2 School of Population Health, University of Queensland, Australia

## Abstract

**Background:**

There is some evidence from a Cochrane review that rehabilitation following spinal surgery may be beneficial.

**Methods:**

We conducted a survey of current post-operative practice amongst spinal surgeons in the United Kingdom in 2002 to determine whether such interventions are being included routinely in the post-operative management of spinal patients.

The survey included all surgeons who were members of either the British Association of Spinal Surgeons (BASS) or the Society for Back Pain Research. Data on the characteristics of each surgeon and his or her current pattern of practice and post-operative care were collected via a reply-paid postal questionnaire.

**Results:**

Usable responses were provided by 57% of the 89 surgeons included in the survey. Most surgeons (79%) had a routine post-operative management regime, but only 35% had a written set of instructions that they gave to their patients concerning this. Over half (55%) of surgeons do not send their patients for any physiotherapy after discharge, with an average of less than two sessions of treatment organised by those that refer for physiotherapy at all. Restrictions on lifting, sitting and driving showed considerable inconsistency both between surgeons and also within the recommendations given by individual surgeons.

**Conclusion:**

Demonstrable inconsistencies within and between spinal surgeons in their approaches to post-operative management can be interpreted as evidence of continuing and significant uncertainty across the sub-speciality as to what does constitute best care in these areas of practice. Conducting further large, rigorous, randomised controlled trials would be the best method for obtaining definitive answers to these questions.

## Background

Whilst low back pain (LBP) affects 60–80% of the population at some point in their lives [1], only 0.5% undergo surgical intervention [2]. However, this inpatient treatment forms the largest single component of overall expenditure related to LBP within the NHS [3]. It is therefore important that such intervention is effective.

The existing literature supports the use of surgical intervention in the management of nerve root stenosis and disc protrusion [4-9]. However, with sizeable and widely varying proportions of patients experiencing recurrent low back and leg pain or other impairment post-operatively, optimising the outcome of spinal surgery appears to be a priority for further research. [5,8,10]. Since many patients have structural abnormalities in their back muscles and poor levels of function [11-16] at the time of surgery, which can be compromised further by the surgery itself [17], improving post-operative care in terms of advice and rehabilitation potentially is an important area to consider.

A review published in 2003 [18] identified thirteen controlled trials that compared an active rehabilitation programme with standard post-operative care in patients undergoing spinal surgery. These studies suggested that post-operative exercise regimes led to a more rapid return to work. Further work by Christensen, published in 2004, supported the concept of rehabilitation but noted the added benefits from group and therapist interactions in the form of a Back Café approach. [19] Whether such interventions are being included routinely in the post-operative management of spinal patients is not clear. Accordingly, we conducted a survey of post-operative practice amongst spinal surgeons in the United Kingdom. The aim of this survey was to obtain an overview of spinal practice rather than details of practice specific to each surgical procedure. At the time our survey was conducted, all of the 13 trials included in the Cochrane review [18] had already been published.

## Methods

Surgeons in the UK who were members of either the British Association of Spinal Surgeons (BASS) or the Society for Back Pain Research were surveyed using an anonymous, two-page, reply-paid questionnaire. This questionnaire was initially piloted amongst 4 spinal surgeons at a large teaching hospital and modifications were made prior to the definitive survey. The final questionnaire sought information about each surgeon's experience, place(s) of work (National Health Service *versus *private hospital), population of patients undergoing surgery, the operations performed, and whether the surgeon had a routine for post-operative management following spinal surgery. Surgeons were questioned regarding whether or not they had a specific postoperative practice for each of the following procedures: discectomy, decompression, and fusion with and without instrumentation, and asked to provide details if available. Further questions regarding specific interventions were kept at a general level. Care was taken not to send surgeons who were members of more than one society multiple questionnaires. Non-responders were sent a further questionnaire after one month. The Riverside Research Ethics Committee approved the protocol for the study.

### Analysis

We analysed replies to the questionnaire using Microsoft Access to construct frequency tables and the statistical programme SPSS to compute rank correlations of recommended post-operative activities.

## Results

Of the 89 questionnaires distributed, 63 (71%) were returned; 51 (57%) could be used in the analysis. All percentages given below are based on these 51 responses. Twelve questionnaires could not be used, primarily due to retirement of the surgeon (n = 7), although some responses were incomplete (n = 5). The surgeons' mean duration of experience was 15.1 years (range: 2–30 years), and they performed an average of 153 operations each year (range: 7–400). They worked in a variety of settings: 18% in specialist centres, 43% in District General Hospitals, 49% in teaching hospitals, and 75% in private practice. Many worked in different combinations of these four settings, with only three active exclusively in private practice. All of the surgeons who responded performed both discectomies and spinal decompression operations, with 61% performing fusion both with and without instrumentation, 12% always using instrumentation during their fusions, 6% never using instrumentation and the remainder not performing fusion surgery. Whilst 47% opted for open surgical procedures, one surgeon undertook minimally invasive surgery only and 51% performed both open and minimally invasive operations.

The majority of surgeons (79%) stated that they had a routine post-operative management regime. However, only 35% had a written set of instructions that they gave to their patients concerning their post-operative management, with a few stating that a written document was under development. None stated that their practice varied depending on the surgical procedure performed. In terms of the initial post-operative period, 70% of surgeons aim to mobilise their patients out of bed on day one post-surgery, 20% on day 2 and 10% leave it until the patient feels able. Care after discharge shows greater variability, although a majority of surgeons arranging the first post-discharge review for six weeks (Table 1).

In terms of post-operative advice and restrictions, the majority of surgeons (73%) discouraged bed rest during the post-operative period. However, 6% recommended additional bed rest during the first week; 12 % up to the second week and 8% encouraged it up to the third week following surgery. Some surgeons imposed restrictions on sitting after their patients were discharged from hospital, with one third (31%) of surgeons requesting that their patients do not sit for between 2 days to 6 weeks. Only a minority of surgeons (18%) recommended use of a corset after a spinal operation; this may be related to those surgeons performing fusion without instrumentation, but was not explored in the questionnaire.

Restrictions of lifting and driving showed the greatest inconsistency between surgeons. A large proportion of surgeons (45%) restricted lifting until the end of the 12^th ^post-operative week, but 20% nominated six weeks and a few did not restrict lifting (Table 1). There was similar variation with respect to proscriptions on driving.

Recommendations with respect to return to work also varied between surgeons and between sedentary and manual work, an average time of ten weeks off work for manual workers compared with around 5 weeks for individuals in sedentary occupations. The average patient was advised to wait 12 weeks until returning to sporting activity, but different surgeons nominated periods between 4 and 28 weeks. Our failure to distinguish between contact sport, other competitive pursuits and lower grades of exercise may have contributed to this variation.

As regards post-operative rehabilitation/physiotherapy, Table 1 reveals that over half (55%) of respondents did not send their patients for any physiotherapy after discharge. For those that did, the number of sessions ranged from 2 to 8 (mean 1.3) for patients in the public sector, whilst those in the private sector could expect slightly more physiotherapy, with the number of sessions ranging from 1 to 10 (mean 1.8).

Table 2 presents rank correlation coefficients summarising the consistency of individual surgeon's recommendations to patients regarding periods in which particular activities should be avoided. Although all are positive, most of the correlations are weak and there is a remarkable inconsistency between recommendations regarding sitting and return to sedentary work.

## Discussion

This study suggests that there is wide variation in practice amongst spinal surgeons with respect to post-operative management. Our relatively modest response fraction of 57% does not affect this finding as the apparent variation could only increase had more surgeons returned our questionnaire. Because no surgeon stated that their practice varied depending on the surgical procedure performed, it seems unlikely that differences between procedures have contributed importantly to the range of practice regarding post-operative management that our survey has revealed. Interestingly, active rehabilitation did not feature prominently in the approach of many surgeons. This is surprising considering that all of the papers included in the Cochrane review [18] had been published before our survey took place and all recommend the inclusion of exercise. Previous surveys highlighted variations with respect to recommendations regarding return to work ranging from 1–16 weeks [7,20]. Similarly, a study by Magnusson *et al *[21] into restrictions on lifting revealed obvious differences in recommendations. Magnusson *et al*. [21] were unable to identify any evidence in the literature to support such restrictions, even when mechanical factors were considered [22]. Our paper now adds evidence of a notable discordance between post-operative restrictions on sitting and recommendations about return to sedentary work or driving.

There is an abundance of evidence indicating significant impairment of muscle function after spinal surgery [12,15,17], suggesting that postoperative rehabilitation might be routine practice. While the available controlled trials comparing an active rehabilitation programme with standard post-operative care in patients undergoing spinal surgery support this inference [18], most of these studies were small (a mean of 72 participants, range 12–212) and the measures of outcome limited, suggesting further work is required to confirm that rehabilitation should indeed be part of routine post-operative care.

A previous study of anaesthetists has revealed that while individual practitioners and even institutions may demonstrate complete uniformity in aspects of peri- and post-operative care, a wider survey reveals very considerable heterogeneity of practice in regard to common operations and conditions [23], a finding substantiated in the orthopaedic literature [24,25] and by the present data. This sometimes is the result of slow uptake of the results of randomised controlled trials but more often it reflects an absence of high quality evidence, uncertainty across the health system as a whole as to the best method for managing a particular clinical situation, and reliance by individuals on what they themselves were taught blended with their clinical impression and experience.

It must be noted that this study focused on surgeons declaring a particular interest in the spine through membership of specialist societies. Their practice may not reflect that of the whole population of surgeons performing spinal operations. However, one would expect that surgeons with a special interest in the spine would be more aware of research findings in the field, particularly the Cochrane review [18], just as specialist breast surgeons, for example, report patterns of practice that are more consistent with guidelines based on research evidence [26]. Broadening the sample of surgeons or increasing the response to our survey are both only likely to reveal greater ranges of practice, even if particular modes of practice appeared relatively more prominent.

## Conclusion

In summary, the optimal post-operative management of patients undergoing spinal surgery may make a significant contribution to improving the long-term outcome of these operations. The evidence presently available from the literature suggests that routine post-operative rehabilitation should form part of such efforts, but the trials of this strategy that have been completed suffer from a number of deficiencies. The basis for restrictions on particular activities in the post-operative period, and especially the recommended periods over which patients should observe such restrictions, is even less clear. At the same time, demonstrable inconsistencies within and between these aspects of different surgeons' practice can be interpreted as evidence of continuing and significant uncertainty across the sub-speciality as to what does constitute best care in these areas of management. Expanding the survey population or increasing the response to the current survey would only highlight this uncertainty further. Conducting further large, rigorous, randomised controlled trials would be the best method for obtaining definitive answers to these questions.

## Competing interests

The author(s) declare that they have no competing interests.

## Authors' contributions

AMcG participated in the design of the study, interpretation of the data and analysis and preparation of the manuscript.

BD participated in the design of the study, and the collection and analysis of the data.

KJ participated in the design of the study, interpretation of the data and analysis and preparation of the manuscript.

## Pre-publication history

The pre-publication history for this paper can be accessed here:



## Figures and Tables

**Table 1:** Summary of responses to questions on post-operative management

**Aspect of management**	**Week 0**	**Week 1–2**	**Week 3–6**	**Week 7–12**	**>Week 12**
Timing of post-operative review	0	10%	79.5%	10.5%	0
Duration of restriction on lifting	15%	0	20%	55%	10%
Duration of restriction on driving	18%	16%	58%	8%	0
Recommended return to sedentary work	8%	12%	72%	8%	0
Recommended return to manual work	8%	0	28%	52%	12%

**Sessions of physiotherapy**	**0**	**1–2**	**3–6**	**7–12**	**>12**

NHS patients	74%	8%	14%	4%	0
Private patients	68%	2%	22%	8%	0

**Table 2:** Spearman correlation coefficients for agreement between duration of restrictions on activity recommended after spinal surgery

	No sitting	No driving	No sedentary work	No lifting	No manual work	No sport
No sitting		0.36	0.08	0.27	0.15	0.36
No driving			0.32	0.37	0.34	0.4
No sedentary work				0.35	0.71	0.20
No lifting					0.39	0.47
No manual work						0.26
No sport						
